# Crosstalk between the tRNA methyltransferase Trm1 and RNA chaperone La influences eukaryotic tRNA maturation

**DOI:** 10.1016/j.jbc.2023.105326

**Published:** 2023-10-06

**Authors:** Jennifer Porat, Ana Vakiloroayaei, Brittney M. Remnant, Mohammadaref Talebi, Taylor Cargill, Mark A. Bayfield

**Affiliations:** Department of Biology, York University, Toronto, Canada

**Keywords:** RNA, tRNA, RNA chaperone, RNA modification, RNA methylation

## Abstract

tRNAs undergo an extensive maturation process involving posttranscriptional modifications often associated with tRNA structural stability and promoting the native fold. Impaired posttranscriptional modification has been linked to human disease, likely through defects in translation, mitochondrial function, and increased susceptibility to degradation by various tRNA decay pathways. More recently, evidence has emerged that bacterial tRNA modification enzymes can act as tRNA chaperones to guide tRNA folding in a manner independent from catalytic activity. Here, we provide evidence that the fission yeast tRNA methyltransferase Trm1, which dimethylates nuclear- and mitochondrial-encoded tRNAs at G26, can also promote tRNA functionality in the absence of catalysis. We show that WT and catalytic-dead Trm1 are active in an *in vivo* tRNA-mediated suppression assay and possess RNA strand annealing and dissociation activity *in vitro*, similar to previously characterized RNA chaperones. Trm1 and the RNA chaperone La have previously been proposed to function synergistically in promoting tRNA maturation, yet we surprisingly demonstrate that La binding to nascent pre-tRNAs decreases Trm1 tRNA dimethylation *in vivo* and *in vitro*. Collectively, these results support the hypothesis for tRNA modification enzymes that combine catalytic and noncatalytic activities to promote tRNA maturation, as well as expand our understanding of how La function can influence tRNA modification.

Owing to their critical role in translation, tRNAs are subject to numerous processing and quality control steps to ensure their structural stability and functionality. In eukaryotes, this involves removal of a 5′ leader and 3′ trailer sequence, where applicable, removal of an intron, CCA addition and aminoacylation of the mature 3′ end, as well as the acquisition of posttranscriptional modifications (reviewed in (1, 2)). While most posttranscriptional modifications are nonessential, especially in yeast, the combination of modifications on a given tRNA is an important determinant for structural stability and functionality. Modifications to the anticodon loop (ACL) affect translational fidelity by maintaining the correct ORF and influencing codon-anticodon base-pairing, whereas modifications to the tRNA body primarily affect structure and folding (3). Importantly, studies have found that pre-tRNAs lacking certain posttranscriptional modifications are prone to misfolding and subsequently targeted for decay by the nuclear surveillance machinery (4, 5), while hypomodified mature tRNAs are degraded by the rapid tRNA decay pathway (6, 7, 8).

Links between pre-tRNA structure and escape from decay are especially relevant in the context of the function of the eukaryotic RNA chaperone La. The La protein interacts with the 3′ uridylate trailer of RNA polymerase III transcripts, including nascent pre-tRNAs, through a binding pocket formed by the eponymous La motif and RNA recognition motif 1 (RRM1) (9, 10, 11, 12). As such, a major role of the La protein is to protect the 3′ end of pre-tRNAs from exonucleolytic degradation and direct the order of pre-tRNA end processing (reviewed in (13)). La binding to the 3′ trailer results in 5′ leader processing by RNase P followed by 3′ trailer cleavage, the latter of which enables La dissociation and recycling onto a new pre-tRNA substrate (14, 15). 3′ end binding by La is particularly important for structurally defective pre-tRNAs, which rely on La for protection from decay by the nuclear exosome (16). However, 3′ end protection alone is insufficient to rescue increasingly defective pre-tRNAs from nuclear surveillance, necessitating a second activity mapping to the canonical RNA binding surface of the RRM1 (15, 16). Further insight into this alternate activity revealed that the RRM1 interacts with the pre-tRNA body, where it can act as an RNA chaperone to assist pre-tRNA folding (15, 17, 18). Coupling of La’s two distinct binding modes—3′ uridylate binding and contacts to the tRNA body—enables high-affinity engagement of pre-tRNAs, resulting in their stabilization and proper folding (15).

As tRNAs can also achieve their native, functional conformation through posttranscriptional modifications, the La protein has been proposed to function redundantly with tRNA modification enzymes. In agreement with this, deletion of La is synthetically lethal with the deletion of several tRNA modification enzymes in budding and fission yeast grown at elevated temperatures, where tRNA misfolding can occur (18, 19). Rescue of synthetic lethality by WT La, but not RRM1 mutants linked to defective RNA chaperone function, further supports the idea that La and tRNA modification enzymes have roles in promoting correct tRNA folding (18). Among the modification enzymes that function redundantly with La, N2, N2-dimethylation at G26 by the tRNA methyltransferase Trm1 (20), has been demonstrated to stabilize pre-tRNA *in vitro* and *in vivo* (18, 21). G26 dimethylation has been linked to structural stability at the junction between the anticodon and variable arm, or hinge region, in that lack of G26 dimethylation results in increased accessibility of nucleotides in the hinge of a pre-tRNA substrate to chemical probing by lead acetate *in vitro* (18). While studies on the structure-stabilizing effect of Trm1-catalyzed methylation have largely been limited to pre-tRNAs in the nucleus, nuclear-encoded Trm1 contains an N-terminal mitochondrial targeting sequence, leading to the modification of select G26-containing mitochondrial-encoded tRNAs (22, 23). The human mitochondrial-localized Trm1 has been linked to promoting protein synthesis, cellular proliferation, and redox homeostasis through modification of mt-tRNA Ile^UAU^, mt-tRNA Ala^UGC^, and mt-tRNA Arg^UCG^ (22). In contrast, budding yeast produce two Trm1 isoforms arising from alternate transcription start sites that yield proteins differing in the presence of the mitochondrial targeting sequence (23). While the additional N-terminal sequence enhances the efficiency of mitochondrial targeting, it is not strictly required for mitochondrial import, as both isoforms are capable of methylating mitochondrial-encoded tRNAs (23).

The structural and functional importance of posttranscriptional modifications on both nuclear- and mitochondrial-encoded tRNAs has been well-established (reviewed in (24)), but recent evidence points to the idea that posttranscriptional modification enzymes may have alternate functions related to tRNA folding (2). Notably, the bacterial tRNA pseudouridine synthase TruB and methyltransferase TrmA promote tRNA folding even in the absence of catalysis, leading to their characterization as tRNA chaperones (25, 26). Although a catalytically inactive mutant of the eukaryotic TrmA homolog Trm2 can rescue a growth defect associated with a mutant allele of tRNA Ser^CGA^, suggesting that the dual function of tRNA modification enzymes is evolutionarily conserved (27), to date there has been no mechanistic insight into how eukaryotic tRNA modification enzymes promote tRNA folding independent from catalytic activity.

Here, we investigated the importance of catalytic *versus* noncatalytic functions of the tRNA methyltransferase Trm1 from *Schizosaccharomyces pombe* and examined crosstalk between Trm1 and function of the *S. pombe* La homolog Sla1, an established RNA chaperone. We confirmed that similar to budding yeast, an N-terminal mitochondrial localization signal enabled select modification of mitochondrial-encoded but not nuclear-encoded tRNAs. We demonstrated that mutation of a key catalytic residue resulted in a complete loss of Trm1 modification at G26 *in vitro* and *in vivo* but that this mutant nevertheless promoted pre-tRNA maturation in a tRNA-mediated suppression assay. We also uncovered the timing of Trm1-mediated dimethylation with respect to other pre-tRNA processing activities and unexpectedly showed that Sla1 opposes tRNA modification by Trm1. Finally, we provided evidence that both WT and catalytically inactive Trm1 are functional in an RNA strand annealing and dissociation assay *in vitro*, suggesting that Trm1 may also act as a tRNA chaperone. These data are thus consistent with the idea that tRNA modification enzymes have retained modification and modification-independent activities throughout evolution and use a combination of these activities to ensure proper tRNA structure and function.

## Results

### Alternate transcriptional start sites yield nuclear- and mitochondrially-targeted Trm1 in *S. pombe*

**Figure 1:**
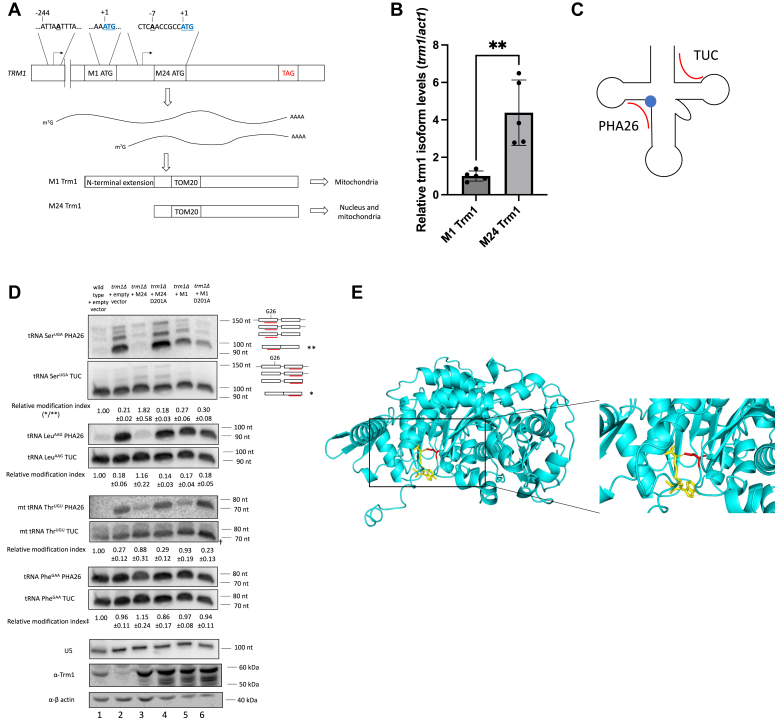
***Schizosaccharomyces pombe* Trm1 modifies nuclear- and mitochondrial-encoded tRNAs at G26.***A*, schematic of alternate transcription start sites giving rise to nuclear- and mitochondrial-targeted Trm1 isoforms. The amphiphilic N-terminal sequence in the mitochondrial-targeted isoform enhances the efficiency of mitochondrial targeting, while both isoforms contain a TOM20-binding site for mitochondrial import. *B*, quantitative reverse transcriptase polymerase chain reaction of *trm1* mRNA isoforms normalized to *act1* mRNA (mean ± standard error, two-tailed unpaired *t* test ∗∗ at *p* < 0.01) (n = 5 biological replicates). *C*, schematic depicting binding sites of the PHA26 and TUC probes (*red*). G26 is represented by a *blue circle*. *D*, PHA26 northern blot of nuclear- and mitochondrial-encoded tRNA. Northern blots were stripped and reprobed for U5 as a loading control. Relative modification index represents the TUC signal divided by the PHA26 signal and normalized to a WT strain (mean ± SEM, n = 3 biological replicates). The relative modification index for tRNA Ser^UGA^ was calculated using the signal corresponding to the mature tRNA (∗∗/∗). The prominent band corresponding to mt tRNA Thr^UGU^ is indicated (†) to differentiate it from a nonspecific background band. ‡Note that although tRNA Phe^GAA^ is not modified, a relative modification index is still provided to demonstrate that the ratio of TUC/PHA26 signal remains unchanged based on the presence of Trm1. *Bottom panels*: Western blot of Trm1 and β-actin in a WT and *trm1Δ* strain transformed with the indicated plasmids. *E*, AlphaFold (30) structure prediction of Trm1 aligned to SAH-bound Trm1 from *Pyrococcus horikoshii* (PDB 2EJU) (31). *Inset*: Hydrogen bonding between SAH (*yellow*) and D201 (*red*).

Trm1 from budding yeast can exist as a nuclear- or mitochondrial-targeted isoform produced from alternate transcription start sites that result in the inclusion or omission of an N-terminal mitochondrial targeting sequence (23). Two alternate transcription start sites have been mapped for fission yeast Trm1: one producing a ∼250 nucleotide 5′ UTR ahead of a more upstream ATG (this isoform will henceforth be referred to as M1, for beginning at the first methionine) and a second with a ∼10 nucleotide 5′UTR ahead of a downstream ATG (which we refer to as M24, beginning at the 24th amino acid) (Fig. 1*A*) (28). While both the longer and shorter isoforms in budding yeast have been shown to target mitochondrially encoded tRNAs, the N-terminal extension in the longer isoform enhances the efficiency of mitochondrial targeting, resulting in a predominantly mitochondrial-localized protein compared to the nuclear-localized shorter isoform (23). Quantification of the relative abundance of both transcripts in *S. pombe* cells grown in rich media revealed that the shorter transcript giving rise to the M24 isoform was approximately four times as abundant as the mitochondrially targeted transcript (M1) at steady state levels (Fig. 1*B*). Consistent with the idea that modifications to the tRNA body do not dramatically alter bulk translation, pulse labeling revealed no defects in mitochondrial translation upon Trm1 deletion (Fig. S1).

To monitor Trm1-catalyzed modification of nuclear- and mitochondrially-encoded tRNAs, we used an established northern blotting–based assay: positive hybridization in the absence of modification at G26 (PHA26) (21, 29), in which dimethylation at G26 impairs hybridization of a probe designed to anneal to the region overlapping G26. A second probe targeting the 3′ end of the tRNA and extending into the TUC stem, which is free of modifications that interfere with probe hybridization, served as an internal normalization for tRNA abundance (Fig. 1*C*). These northern blots supported the expected subcellular targeting of the Trm1 isoforms: M24 Trm1 robustly modified nuclear- and mitochondrial-encoded tRNAs, consistent with its proposed localization to the nucleus and mitochondria, while M1 Trm1 only modified mitochondrially-encoded tRNAs, suggesting that it is solely present in the mitochondria (Fig. 1*D*, lane 5). The ability of overexpressed M24 to rescue mitochondrial tRNA modification levels to a similar degree as overexpressed M1 may result from the increased expression of plasmid-encoded Trm1 relative to endogenous Trm1 (Fig. 1*D*, lane 3), leading to an increase in the amount of Trm1 capable of entering the mitochondria.

These results also confirmed the previously demonstrated substrate specificity of Trm1, where not all G26-containing tRNAs are Trm1 substrates (21). Certain nuclear-encoded tRNAs, including tRNA Ser^UGA^ and tRNA Leu^AAG^, were robustly modified by endogenous Trm1 (Fig. 1*D*, “wild type”, lane 1) and overexpressed M24 Trm1 (Fig. 1*D*, lane 3), while the PHA26 probe annealed to the G26-containing tRNA Phe^GAA^ in a manner that remained unchanged based on the presence of Trm1, consistent with a lack of modification (see figure caption for additional details on quantification of relative modification levels).

### D201 is a key catalytic residue for N2, N2-dimethylation of nuclear- and mitochondrially-encoded tRNAs

To investigate potential modification-independent functions of Trm1, we aligned an AlphaFold (30) prediction of *S. pombe* Trm1 to the structure of an archaeal Trm1 homolog bound to SAH (31), mutated a conserved aspartic acid directly in the predicted catalytic site to alanine (D201A) (Fig. 1*E*), and again monitored Trm1-catalyzed modification of nuclear- and mitochondrially-encoded tRNAs by the PHA26 assay. As expected from the proposed role of this conserved aspartic acid in deprotonating G26 for nucleophilic attack of SAM (31), the D201A mutant showed the same lack of modification of nuclear- and mitochondrial-encoded tRNAs as *trm1Δ* cells transformed with an empty vector, despite similar levels of protein accumulation as the WT-overexpressed isoform (Fig. 1*D*).

**Figure 2:**
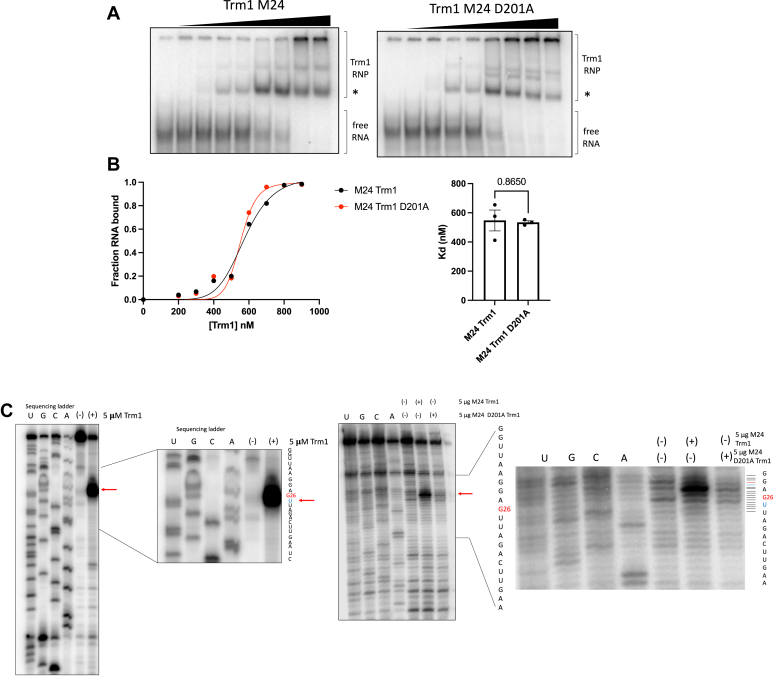
**D201A supports *in vitro* tRNA binding, but not methylation.***A*, representative EMSAs of Trm1 M24 and M24 D201A with radiolabeled pre-tRNA Ser^UGA^. The *asterisk* represents the initial binding event. *B*, representative binding curves (*left*) and *K*_D_ values of (*A*) (*right*) (mean ± SEM, two-tailed unpaired *t* test, n = 3 technical replicates) (see Fig. S2 for all binding curves and Table S5 for individual Kd values and Hill coefficients). *C*, primer extension of *in vitro*–methylated tRNA Ser^UGA^ with 5 μM (*left*) or 5 μg (*right*) recombinant Trm1. Note that while sequencing lanes were constructed with ddNTPs, the ribonucleotide corresponding to the tRNA sequence is indicated in the lane labels. The RNA sequences flanking G26 are indicated and the modification-dependent RT stop, which occurs one nucleotide 3′ of G26 (U27, in *blue*), is indicated by an *arrow*.

To rule out defects in tRNA binding contributing to a lack of modification by D201A, we purified recombinant WT and D201A M24 Trm1 and measured *in vitro*–binding affinity to pre-tRNA Ser^UGA^ using EMSAs. WT and D201A exhibited comparable binding affinity, suggesting that disruption of the putative catalytic site does not impair tRNA-binding affinity or binding cooperativity (Figs. 2, *A* and *B* and S2, Table S5). We note that the supershift binding patterns (defined as complexes migrating at a higher molecular weight than the initial binding event indicated with an asterisk) and cooperativity of binding (as evident by the Hill coefficients in Table S5) were slightly different between WT Trm1 and D201A. We anticipate that the initial binding event represents the physiologically relevant binding event, while the additional bound conformations at higher concentrations may result from more than one protein bound per RNA at sites that are not the primary binding site. The presence of multiple supershift bands with the D201A mutant may be therefore due to unanticipated differences in substrate accommodation occurring upon nonphysiologically relevant binding events when one tRNA is bound by multiple proteins.

We also set up *in vitro* methylation reactions with pre-tRNA Ser^UGA^, recombinant Trm1, and SAM and measured modification efficiency by primer extension, as dimethylation on the Watson-Crick face is sufficient to cause a reverse transcriptase stop (18, 32). The lack of modification by D201A *in vitro* (Fig. 2*C*), evident by the lack of reverse transcriptase stop, and by PHA26 northern blotting *in vivo* (Fig. 1*D*) confirmed that D201A is indeed catalytically inactive, thus enabling further studies into potential catalytic-independent functions of Trm1.

### Trm1 promotes tRNA-mediated suppression through catalytic and catalytic-independent activities

tRNA-mediated suppression is an established system in *S. pombe* that has been used to test various aspects of pre-tRNA maturation including 5′ leader, 3′ trailer and intron processing, and tRNA modification (reviewed in (33, 34)). The assay relies on a mutation to the anticodon of tRNA Ser^UCA^, allowing stop codon readthrough of a nonsense mutation in the AIR carboxylase gene which, when fully functional, prevents the accumulation of a red metabolic intermediate (33, 35). The G35C mutation that enables nonsense decoding also impairs anticodon-intron base pairing in the suppressor pre-tRNA, resulting in a misfold that increases susceptibility of the pre-tRNA to nuclear pre-tRNA quality control and exosome-mediated decay (16). Thus, this assay has been used previously to monitor pre-tRNA susceptibility to nuclear surveillance (16). Suppressor pre-tRNA degradation linked to mutations predicted to cause misfolding can be rescued by the overexpression of pre-tRNA processing factors, enabling identification and insight into the mode of action of pre-tRNA–binding proteins, including the RNA chaperone La (15, 16). We and others have previously demonstrated that Trm1 is active in promoting tRNA-mediated suppression (18, 21), although this could be due to a stabilizing effect from the modification, a separate pre-tRNA maturation or stabilizing activity that functions independently from modification, or a combination of these.

**Figure 3:**
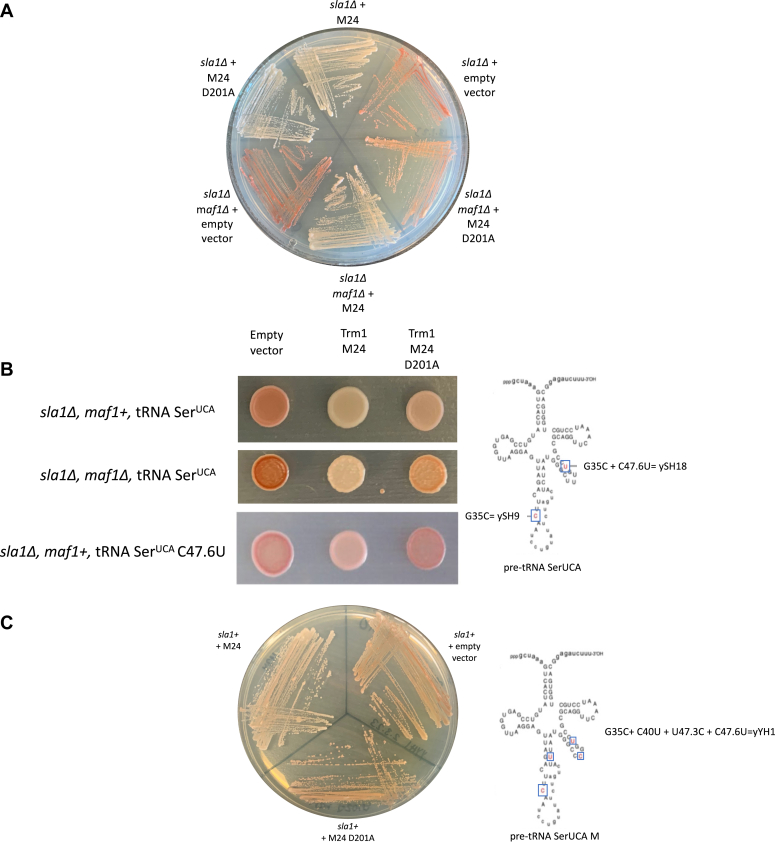
**Trm1 promotes tRNA-mediated suppression independent of catalytic activity.***A*, tRNA-mediated suppression with WT and catalytically inactive Trm1 in a *sla1Δ* and *sla1Δ maf1Δ* background. *B*, tRNA-mediated suppression with WT and catalytically inactive Trm1 in a *sla1Δ* and *sla1Δ maf1Δ* background with increasingly defective suppressor tRNA alleles. *Right*: schematic of the G35C (yeast strain ySH9) and G35C C47.6U (yeast strain ySH18) suppressor tRNA alleles. *C*, tRNA-mediated suppression of the MSer suppressor tRNA allele with WT and catalytically inactive Trm1 in a *sla1+* strain. *Right*: Schematic of the MSer suppressor tRNA allele integrated into the yeast strain yYH1.

We found that both WT and catalytically inactive nuclear-targeted (M24) Trm1 promoted suppression of the tRNA Ser^UCA^ allele in a *sla1Δ* background, suggesting that modification is not strictly required for suppression activity and that Trm1 promotes pre-tRNA maturation even in the absence of catalysis (Fig. 3, *A* and *B*). Addition of the U47:6C (yeast strain ySH18) or C40U, U47.3C, and C47.6U (yeast strain yYH1) mutations, which are associated with increased reliance on the RNA chaperone activity of La (17, 36), resulted in partial tRNA-mediated suppression by WT Trm1 but not D201A (Fig. 3, *B* and *C*). These data are suggestive of two activities for Trm1: its established methyltransferase activity, which has been previously shown to stabilize tRNA structure (18), and a modification-independent activity. The catalytic-independent function is sufficient for suppression activity in the context of the tRNA Ser^UCA^ allele, whereas both activities are required for more defective suppressor tRNA alleles.

It has also been reported that deletion of the RNA polymerase III repressor Maf1 causes antisuppression in the tRNA-mediated suppression assay, which at first was noted to be counter-intuitive with the increase in tRNA transcription observed upon Maf1 deletion (21, 37). However, this antisuppression phenotype can be explained by hypomodification of the suppressor tRNA by Trm1, as Trm1—and presumably other pre-tRNA–binding proteins— become limiting with the resultant increase in the pre-tRNA pool (21). We found that suppression of tRNA Ser^UCA^ is achieved by WT and catalytically inactive Trm1 in the *maf1Δ* strain, although we note that the degree of suppression, as determined by a lack of red pigment accumulation, was greater for WT Trm1 than the catalytically inactive mutant (Fig. 3, *A* and *B*). In the model whereby Trm1 becomes limiting in the absence of Maf1, suppression by D201A suggests that it is not only the Trm1-catalyzed modification that becomes limiting but also a catalytic-independent function for Trm1. Still, the additional suppression achieved by WT Trm1 compared to the catalytically inactive mutant supports a stronger role for the Trm1-catalyzed modification than a secondary catalytic-independent activity.

**Figure 4:**
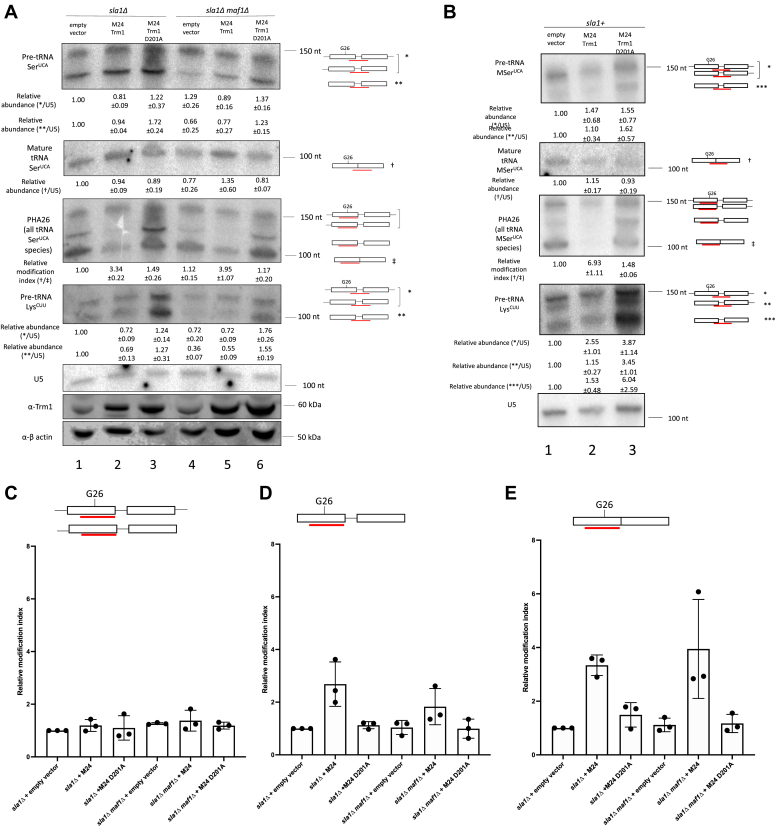
**Trm1 modifies end-processed, intron-containing pre-tRNAs.***A*, northern blot of tRNA Ser^UCA^ processing intermediates and G26 modification status and 3′ end protection of pre-tRNA Lys^CUU^ and Western blot of Trm1 and β-actin in a *sla1Δ* and *sla1Δ maf1Δ* strain transformed with the indicated plasmids. ∗ refers to the nascent and 3′ end-processed suppressor pre-tRNA, ∗∗ is the intron-containing, end-processed suppressor pre-tRNA, † is the probe overlapping the splice junction, and ‡ is the PHA26 probe overlapping G26 (mean ± SEM, n = 3 biological replicates). This blot was stripped and reprobed for additional tRNA species in Fig. S4 and shares the U5 loading control (lanes 1–3) as in Fig. S4. *B*, northern blot of tRNA Ser^UCA^ processing intermediates and G26 modification status and 3′ end protection of pre-tRNA Lys^CUU^ in a *sla1+* strain transformed with the indicated plasmids. ∗ refers to the nascent and 3′ end-processed suppressor pre-tRNA, ∗∗ refers to the intron- and trailer-containing suppressor pre-tRNA, ∗∗∗ is the intron-containing, end-processed suppressor pre-tRNA, † is the probe overlapping the splice junction, and ‡ is the PHA26 probe overlapping G26 (mean ± SEM, n = 3 biological replicates). This blot was stripped and reprobed for additional tRNA species and shares the U5 loading control as in Fig. S4. *C*–*E*, quantification of relative modification at G26 in (*A*), relative to the *sla1Δ* strain expressing an empty vector. Relative modification was calculated by normalizing the PHA26 signal (overlapping G26) to probes targeting the intron-containing precursor (*C* and *D*) and mature suppressor (*E*) (mean ± SEM, n = 3 biological replicates).

To further investigate how Trm1 promotes tRNA-mediated suppression, we examined suppressor tRNA processing and modification by northern blotting (Fig. 4*A*). Probes against the tRNA Ser^UCA^ intron were used to detect the precursor, which migrated as two bands in the absence of the yeast La protein Sla1: a leader-containing species that migrated as a higher molecular weight smear due to 3′ exonucleolytic nibbling (represented by ∗ for pre-tRNA Ser^UGA^ and pre-tRNA Lys^CUU^) and a leader- and trailer-processed species (∗∗) (Fig. 4*A*). In contrast, the presence of Sla1 resulted in three visible pre-tRNA processing intermediates corresponding to the nascent leader- and trailer-containing pre-tRNA (∗), the leader-processed and trailer-containing pre-tRNA (∗∗), and the fully end-processed pre-tRNA (∗∗∗) (Fig. 4*B*, pre-tRNA Lys^CUU^). We also used a probe overlapping the exon junction to detect the mature suppressor tRNA. As the probe targeting the exon junctions overlaps the ACL, which contains posttranscriptional modifications such as m^3^C32, i^6^A37, and t^6^A37 which are known to influence probe binding (29, 38, 39), we designed a probe targeting the ACL of WT tRNA Ser^UGA^ to test whether the presence of Trm1 impacts certain ACL modifications (Fig. S3). We did not observe any differences in probe binding due to the presence or absence of endogenous or overexpressed Trm1, suggesting that Trm1 does not influence ACL modifications that are sensitive to northern blotting.

**Figure 5:**
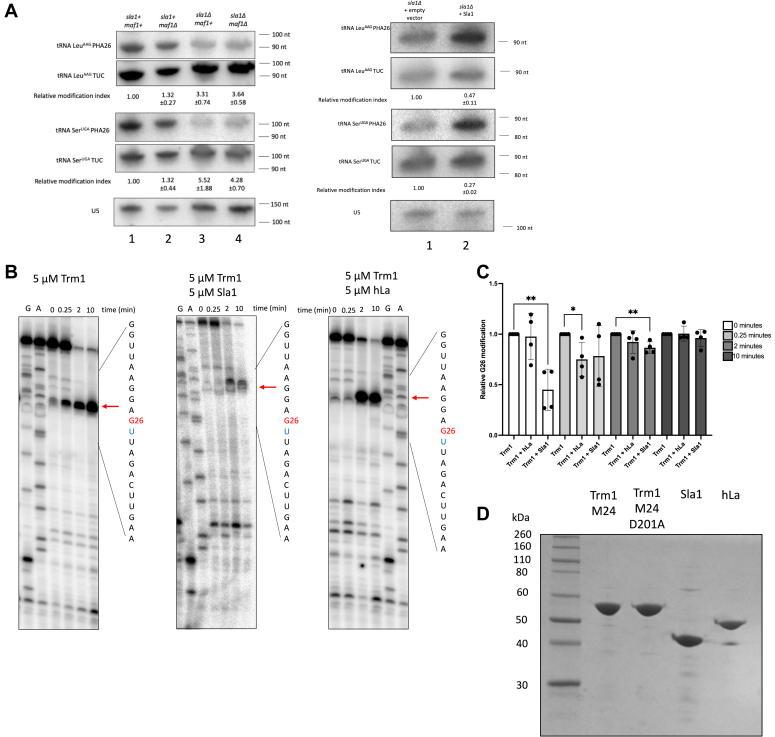
**La and Trm1 influence pre-tRNA end-processing and G26 dimethylation.***A*, PHA26 northern blots of nuclear-encoded tRNA in WT, *maf1Δ*, *sla1Δ*, and *sla1Δ maf1Δ* cells (*left*), and *sla1Δ* strains transformed with an empty vector or Sla1 (*right*). Northern blots were stripped and reprobed for U5 as a loading control. Relative modification index represents the TUC signal divided by the PHA26 signal and normalized to a WT or empty vector–expressing strain (mean ± SEM, n = 3 biological replicates) *B*, primer extension of *in vitro*–methylated tRNA Ser^UGA^ pre-incubated with no protein, *Schizosaccharomyces pombe* La (Sla1), or human La (hLa) prior to incubation with Trm1. *Red* arrows indicate the reverse transcriptase stop corresponding to G26. The RNA sequences flanking G26 are indicated and the modification-dependent RT stop, which occurs one nucleotide 3′ of G26 (U27, in *blue*), is indicated by an *arrow*. The same sequencing ladder was used for Trm1 and Trm1 + hLa. *C*, quantification of *in vitro* methylation of tRNA Ser^UGA^ over time, calculated as the ratio of the G26 RT stop to full length tRNA, relative to the reaction with Trm1 alone (mean ± SEM, , two-tailed unpaired *t* test ∗ at *p* < 0.05, ∗∗ at *p* < 0.01, n = 4 independent replicates). *D*, SDS-PAGE analysis of recombinant proteins used in this study. One microgram of each protein was assessed for quantity and purity and visualized with Coomassie blue stain.

**Figure 6:**
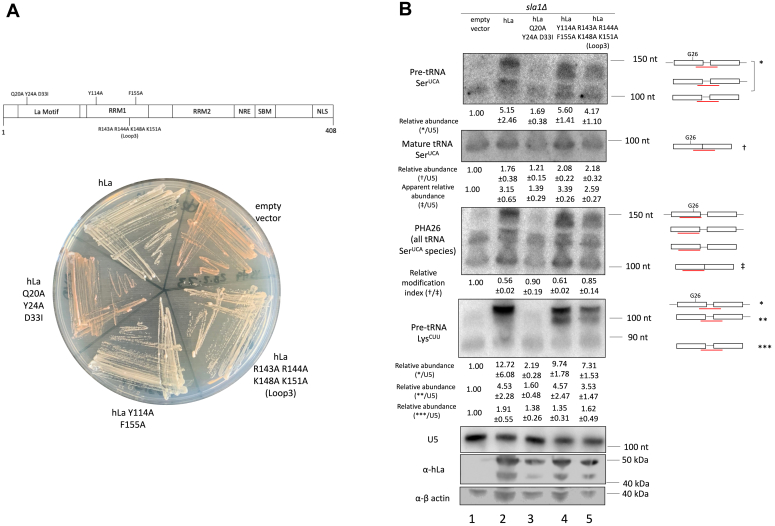
**Crosstalk between La and Trm1 is linked to La-pre-tRNA–binding modes.***A*, schematic of human La (hLa) domains with indicated mutations and tRNA-mediated suppression of WT and mutant hLa constructs in a *sla1Δ* background. *B*, northern blot of tRNA Ser^UCA^-processing intermediates and G26 modification status and 3′ end protection of pre-tRNA Lys^CUU^. Apparent relative abundance refers to intensity of the PHA26 probe normalized to U5. A Western blot for human La and β-actin is provided below. ∗ refers to the nascent and 3′ end-processed suppressor pre-tRNA, ∗∗ refers to the intron- and trailer-containing suppressor pre-tRNA, ∗∗∗ is the intron-containing, end-processed suppressor pre-tRNA, † is the probe overlapping the splice junction, and ‡ is the PHA26 probe overlapping G26. Apparent relative abundance was calculated by dividing signal intensity from the PHA26 probe targeting the mature suppressor tRNA to signal intensity of U5, while relative modification index was calculated by dividing signal intensity of the TUC probe targeting the mature suppressor tRNA divided by the PHA26 probe targeting the mature suppressor tRNA (mean ± SEM, n = 3 biological replicates).

tRNA-mediated suppression of tRNA Ser^UCA^ by human or *S. pombe* La is largely due to 3′ trailer binding and protection of the suppressor pre-tRNA from exosome-mediated decay, which is characterized by the stabilization of a 3′ trailer-containing suppressor pre-tRNA species with ectopic La expression in an *sla1Δ* strain relative to an empty vector (15, 16) (also see Figs. 5 and 6). 3′ end protection by La and La-related proteins is typically monitored by looking for stabilization of the 3′ trailer-containing pre-tRNA Lys^CUU^, due to the increased resolution of pre-tRNA–processing intermediates relative to pre-tRNA Ser^UGA^ (16, 40, 41). tRNA Lys^CUU^ is also modified by Trm1, making it an ideal target to test whether Trm1 functions in tRNA-mediated suppression through 3′ end protection (21). As might be expected based on previous work linking recognition of tRNAs by Trm1 to the D-loop (42), we observed no differences in 3′ end protection upon addition of WT Trm1, as evident by the lack of stabilization of the 3′ trailer-containing species relative to the empty vector (Fig. 4*A*, pre-tRNA Ser^UCA^ and endogenous pre-tRNA Lys^CUU^, compared to stabilization of the nascent pre-tRNA with Sla1, see below). This suggests that unlike the La protein, WT Trm1 promotes tRNA-mediated suppression in the absence of 3′ end protection, much like what has been described for tRNA-mediated suppression by La-related proteins (41). However, we noted that stabilization of the 5′- and 3′-processed pre-tRNA species occurs upon overexpression of D201A, which supports the idea that in the absence of catalysis, Trm1 may remain bound to and stabilize the pre-tRNA (Fig. 4*A*, lanes 3 and 6; Fig. 4*B*, lane 3). The accumulation of pre-tRNA following overexpression is also consistent with our *in vitro* results suggesting that the D201A mutation does not significantly impair Trm1’s tRNA-binding activity (Figs. 2, *A* and *B* and S2), as a loss of high affinity tRNA binding would not be expected to manifest as a pre-tRNA accumulation phenotype, although we note that we have not investigated how other factors involved in pre-tRNA processing might be directly or indirectly affected by D201A overexpression.

We also performed the PHA26 assay to investigate the timing of Trm1 modification in tRNA-mediated suppression (Fig. 4, *A* and *B*, third panel). We observed that overexpression of Trm1 in the *trm1+* suppressor-tRNA–containing strain resulted in a decrease in signal intensity with the PHA26 probe, consistent with previous reports that endogenous Trm1 levels are insufficient for full tRNA modification (21). However, overexpression of the catalytically inactive D201A mutant resulted in comparable modification levels in this strain to an empty vector control (Fig. 4*A*, lanes 3 and 6, Fig. 4*B*, lane 3). The PHA26 assay also provided information on the timing of Trm1 modification with respect to pre-tRNA–processing activities: we observed no change in PHA signal intensity upon Trm1 expression in the trailer-processed, leader-containing pre-tRNA (Fig. 4*A*, top band in third panel), suggesting that it is not a substrate for modification but noted a decrease in signal intensity for the leader- and trailer-processed pre-tRNA and mature tRNA, supporting a previously proposed model that Trm1 modification occurs after pre-tRNA end processing (18) (Fig. 4, *C*–*E*). Still, although Trm1 modification starts to occur at the pre-tRNA level, tRNAs were not fully modified (which we infer from the greater decrease in PHA26 signal intensity between the empty vector and overexpressed Trm1) until the mature tRNA stage. We noted the same pattern, with modification evident on the end-processed, intron-containing pre-tRNA but not the nascent pre-tRNA, for the endogenous tRNA Ser^UGA^ in a WT and *trm1Δ* strain (Fig. 1*D*).

As Sla1 promotes 5′ leader processing prior to 3′ trailer processing, resulting in the accumulation of a 3′ trailer-containing pre-tRNA species (14), we repeated these experiments in a WT (*sla1+*) strain to uncouple Sla1-dependent changes in tRNA 3′ end processing from Trm1-mediated stabilization. For these experiments, we used the MSer suppressor tRNA allele (Fig. 3*C*) (40), which has more mutations predicted to cause misfolds than the G35C and U47:6C alleles and for which endogenous Sla1 levels are insufficient for tRNA-mediated suppression (40). In this *sla+* background, only WT Trm1 was capable of suppressing MSer, much like the U47:6C allele (Fig. 3*C*), supporting the importance of G26 dimethylation in imparting additional tRNA stability for more substantially misfolded tRNAs. Aside from increased accumulation of 3′ trailer-containing pre-tRNA species (Fig. 4*B*, top two bands of pre-tRNA Lys^CUU^), we observed similar patterns to the *sla1Δ* strain: suppression by WT Trm1 was not due to increased 3′ end protection and Trm1 modification occured on end-processed, intron-containing pre-tRNAs (Fig. 4*B*, PHA26). These data support the idea that Trm1-catalyzed modification and activity in the tRNA-mediated suppression assay are not affected by the order of 3′ end processing, as it remains unchanged based on the presence of Sla1.

### The La protein decreases Trm1-catalyzed modification

While previous reports indicate that tRNAs become hypomodified at G26 upon Maf1 deletion (21), we did not observe any modification differences with and without endogenous Maf1 (Fig. 4, *A*, *C*–*E*). Since those previous measurements were performed in a *sla1+* strain and our data are from a *sla1Δ* strain, we examined the relationship between Sla1- and Trm1-dependent modification. Strikingly, we detected a substantial increase in G26 modification upon Sla1 deletion for leucine and serine tRNAs, suggesting a possible crosstalk between Sla1 and Trm1 for pre-tRNA binding (Fig. 5*A*). Importantly, re-expression of Sla1 in an *sla1Δ* strain was sufficient to decrease G26 modification (Fig. 5*A*, right panel), while overexpression of WT Trm1 in a *sla1+* strain increased G26 modification, confirming previous results that endogenous Trm1 is insufficient to fully modify the cellular pool of tRNAs (21) (Fig. 4*B* PHA26 panel, Fig. S4). Conversely, overexpression of Sla1 in a *sla1+* strain resulted in no changes in G26 modification, possibly because endogenous Sla1 levels already outcompete endogenous Trm1 levels (43, 44), with increased Sla1 expression exerting negligible effects on tRNA processing and modification (Fig. S5). Notably, the degree to which G26 modification increased upon Trm1 overexpression was greater in a *sla1+* strain than a *sla1Δ* strain, suggesting that a larger fraction of tRNAs are already fully modified in the absence of Sla1 (Fig. S4).

To investigate whether La binding shields pre-tRNAs from Trm1 modification of nuclear-encoded pre-tRNAs, we pre-incubated pre-tRNA with and without recombinant Sla1 or human La (hLa) and set up an *in vitro* methylation time course (with 0 min representing the time between adding Trm1 and removing an aliquot to the quenching solution) to examine changes in G26 dimethylation. We observed slightly decreased methylation in the presence of Sla1 particularly at earliest time points in the reaction (Fig. 5, *B* and *C*, compare timepoint t = 0 min between gels), consistent with our *in vivo* data demonstrating that La decreases Trm1 modification. While our data with hLa trends towards an inhibitory effect on modification (see timepoint t = 0.25 min), the extent of the effect remains ambiguous in the absence of further kinetic analyses to determine the rate of modification with and without La. As the methylation reaction occurs in the absence of any cellular factors that promote tRNA processing, including other modification enzymes and end-processing endo and exonucleases, this is suggestive of La itself acting as a barrier to Trm1-mediated methylation, rather than decreasing dimethylation indirectly, through its roles in facilitating pre-tRNA processing.

As the La protein has been reported to make multiple contacts to pre-tRNAs—engagement of the uridylate trailer and pre-tRNA body (15, 16, 45)—we took advantage of previously characterized mutants of hLa to map inhibition of tRNA modification to La’s various domains and RNA-binding modes (Fig. 6*A*). We measured tRNA-mediated suppression activity, steady-state precursor and mature tRNA levels, and G26 modification status for the suppressor tRNA Ser^UCA^ in an *sla1Δ* strain transformed with WT or mutant hLa (Fig. 6). As has been previously reported, mutations to uridylate-binding residues in the La motif (Q20A Y24A D33I) led to decreased suppression activity. In contrast, mutations to the RRM1 (Y114A F155A and RRM1 loop-3 R142A R143A K148A K151A), which result in impaired RNA chaperone activity but unchanged uridylate binding (15), exhibited no defects in the tRNA-mediated suppression assay, in agreement with the idea that tRNA Ser^UCA^ predominantly requires 3′ end protection, but not RNA chaperone activity, for complete suppression (16) (Fig. 6*A*). This was evident from stabilization of the top suppressor tRNA band on the northern blots, corresponding to the nascent 5′ leader- and 3′ trailer-containing pre-tRNA species, for WT and RRM1 hLa mutants (Fig. 6*B*).

We observed a robust decrease in G26 modification of the suppressor tRNA in strains transformed with WT hLa and the RRM1 mutant Y114A F155A, which displays decreased RNA chaperone activity but has no pre-tRNA–binding defects (16). On the other hand, we observed no differences in modification between the empty vector and the uridylate-binding mutant and a modest decrease in modification compared to the empty vector by the RRM1 loop-3 R142A R143A K148A K151A mutant, which is defective in RNA chaperone activity and has impaired but not ablated pre-tRNA binding that is not attributed to La’s uridylate-binding mode (15, 17) (Fig. 6*B*). These results suggest that crosstalk between La and Trm1 modification correlates with La’s pre-tRNA binding mode, with mutations that disrupt uridylate or tRNA body binding, which contribute additively to pre-tRNA affinity (15), leading to increased modification at G26. Notably, these results also revealed that northern blot–based measurements of hLa-dependent increases in mature suppressor tRNA levels could arise from a combination of changes in mature tRNA levels and differences in G26 modification, for tRNA probes that overlap with this site. Consistent with this, changes in apparent mature suppressor tRNA levels in the presence or absence of WT or mutant hLa were less pronounced when using a probe that does not overlap with G26 (Fig. 6*B*, compare relative mature tRNA Ser^UCA^ to apparent relative mature tRNA Ser^UCA^). Thus, mature suppressor tRNA levels are not the sole determinant that can be used to predict or assign meaning to tRNA-mediated suppression activity, providing additional evidence for the hypothesis that an increase in specific tRNA activity due to modification or folding is linked to tRNA-mediated suppression activity, rather than suppression due solely to suppressor tRNA accumulation.

### Trm1 promotes RNA strand annealing and dissociation *in vitro*

**Figure 7:**
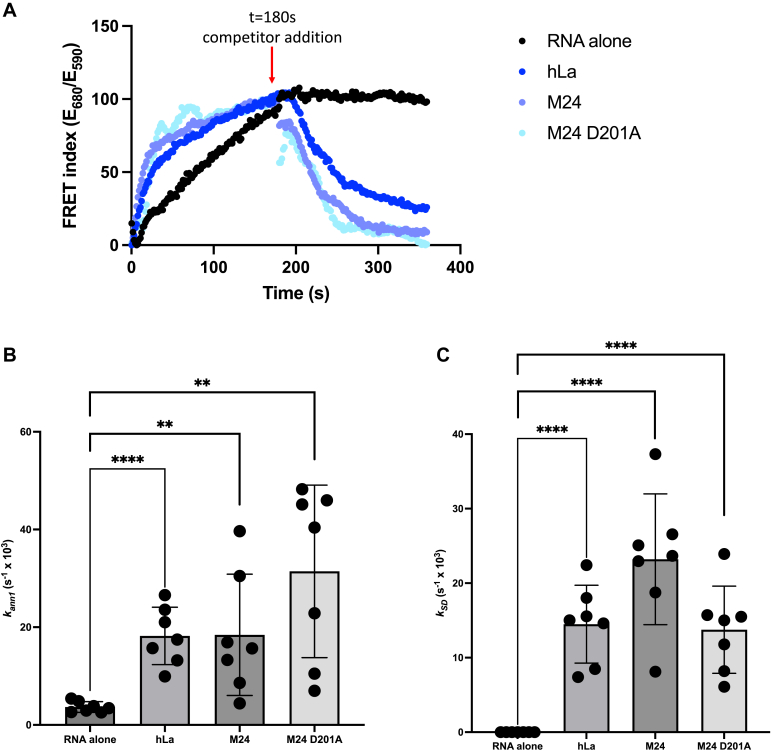
**Trm1 exhibits RNA strand annealing and dissociation *in vitro*.***A*, RNA strand annealing (phase I) and dissociation (phase II), as indicated by changes in FRET index (emission at 680 nm/emission at 590 nm) between the Cy3- and Cy5-labeled substrates. Phase II was initiated with the addition of an unlabeled competitor RNA at t = 180 s. Representative traces are shown. *B* and *C*, strand annealing (*B*) and dissociation (*C*) rate constants calculated with RNA alone, recombinant hLa, or WT or catalytically inactive M24 Trm1 (mean ± SEM, two-tailed unpaired *t* test ∗∗ at *p* < 0.01, ∗∗∗∗ at *p* < 0.0001) (n = 7 independent replicates).

As our data point towards Trm1 possessing a catalytic-independent function that promotes tRNA functionality *in vivo*, we considered the possibility that Trm1 might function as an RNA chaperone, much like the bacterial tRNA-modifying enzymes TruB and TrmA (25, 26). We employed a previously established, *in vitro* FRET-based RNA annealing and dissociation assay that has been used to characterize RNA chaperone activity for La, La-related proteins and other established RNA chaperones such as Hfq and StpA (17, 41, 46, 47, 48). This assay uses complementary RNA oligos 5′ end labeled with Cy3 or Cy5, such that annealing of the oligos results in FRET between the two fluorophores, from which a rate of annealing is calculated (*k*_*ann*_), followed by the addition of excess unlabeled competitor to assess strand dissociation activity (*k*_*SD*_), which is accompanied by a decrease in FRET between the two fluorophores (Fig. 7*A*). In the absence of strand dissociation, as measured by an increase in FRET following competitor addition, the rate constant calculated in the second phase is considered *k*_*ann2*_. Consistent with previous data (17, 41), strand annealing and strand dissociation were enhanced by the addition of recombinant human La (Fig. 7, *B* and *C*). We also observed strand annealing and dissociation for both WT and catalytically inactive Trm1 (Fig. 7, *B* and *C*). Our *in vivo* data demonstrating that Trm1 is active in a tRNA-mediated suppression assay independent of catalysis, coupled with our *in vitro* data supporting an RNA chaperone-like activity, suggest that Trm1 may influence pre-tRNA maturation through a combination of structure-stabilizing modifications and protein-mediated RNA annealing and unwinding, similar to prokaryotic tRNA modification enzymes (25, 26).

## Discussion

In this work, we investigated the interplay between the tRNA modification enzyme Trm1 and the established RNA chaperone La and provided evidence for a catalytic-independent function of Trm1 with functional overlap with genuine La proteins. Similar to La, we anticipate that an RNA chaperone activity linked to a catalytic-independent function of Trm1 may promote pre-tRNA folding in the nucleus, while Trm1-catalyzed dimethylation is particularly important for additional stabilization to resolve more severe misfolds. This model is consistent with our observations comparing tRNA-mediated suppression of various suppressor tRNA mutants: the G35C suppressor tRNA mutation (Ser^UCA^) only results in a misfold at the pre-tRNA level, due to altered base-pairing with the intron which is later removed and so the mature suppressor tRNA possesses the same fold as endogenous tRNA Ser^UGA^. In contrast, adding the U47:6C or C40U, U47.3C, and C47.6U mutations lead to a misfold that persists in the mature tRNA in the cytoplasm, compounded by the fact that the degree of misfold in U47:C or C40U, U47.3C, and C47.6U is also greater than that of tRNA Ser^UCA^. Therefore, while the catalytic-independent activity alone is sufficient for Trm1-associated tRNA Ser^UCA^ stabilization and folding in the nucleus, more defective suppressor tRNA alleles may require both Trm1-mediated pre-tRNA stabilization in the nucleus and the Trm1-catalyzed modification that persists on the mature tRNA in the cytoplasm for full suppression activity (Fig. 3).

We have previously noted a slight charging defect on the endogenous parent of the tRNA Ser^UCA^ suppressor tRNA (tRNA Ser^UGA^) in the context of Trm1 deletion, as well as altered tRNA structure by *in vitro* chemical probing in the absence of G26 modification (18), consistent with tRNA misfolds linked to mature tRNA function being influenced by G26 dimethylation. Given the overlap in cellular localization and apparent functional and genetic crosstalk with the nuclear La protein, we favor a model in which a Trm1 RNA chaperone function associated with the Trm1 D201A mutant may help protect pre-tRNAs from nuclear surveillance, similar to La. In such a scenario, the act of Trm1-binding pre-tRNAs (in the context of modification or a modification-independent activity) might serve to protect the pre-tRNA from nuclear degradation, in an analogous manner to how La binding to the 3′ uridylate trailer prevents nuclear surveillance or EF1A competes with the rapid tRNA decay machinery in the cytoplasm for access to mature tRNAs to prevent their degradation (16, 49). Still, it remains to be found what proportion of pre-tRNAs benefit from Trm1-catalyzed modification *versus* the catalytic-independent function and how Trm1-mediated pre-tRNA stabilization influences downstream tRNA maturation activities to promote tRNA stability and function in translation.

One unexpected result was the apparent inhibition of tRNA modification at G26 by the presence of Sla1, which we validated *in vitro* showing that Sla1 and hLa restrict access of Trm1 to a tRNA substrate. Previously, Trm1 and La were hypothesized to function redundantly in the tRNA biogenesis pathway (18, 19), with La-mediated pre-tRNA stabilization thought to increase the window in which Trm1 could modify pre-tRNAs in the nucleus (18). While it is indeed likely that Trm1 and La have redundant functions—both promote pre-tRNA stability and folding—it is interesting that Sla1 can inhibit access of pre-tRNAs to Trm1. Since both factors promote tRNA function but Sla1 impedes Trm1, it would thus be interesting to speculate about possible situations (stresses, cellular growth conditions) in which a modification at G26 is of greater benefit relative to a tRNA that accesses La-associated RNA chaperone activity. It remains to be found whether a relationship between La and Trm1 exists in humans, where La proteins harbor an additional RNA recognition motif (RRM2 or xRRM) within a more divergent C-terminal domain that has been implicated in La’s RNA chaperone activity (50, 51, 52). The extent to which the C-terminal domain influences tRNA binding *in vivo* remains unknown, although its propensity for sequence-independent binding to structured, hairpin-containing RNA (51, 53) may affect how La engages the pre-tRNA body. Further, exploring how La may influence the installation of other modifications occurring at the pre-tRNA stage will likely be an active area of future research.

Another unexpected but related result concerns the link between apparent mature suppressor tRNA levels and tRNA-mediated suppression activity (16). We found that apparent increases in mature suppressor tRNA levels can at least be partially attributed to a decrease in modification at G26. As relative pre-tRNA levels are often quantitated using probes that anneal to the pre-tRNA intron, a probe that also overlaps with the nearby G26 base might then be reporting on a combination of both pre-tRNA abundance and G26 modification levels, supporting the idea of exercising caution when designing northern blotting probes to avoid sites of bulky modifications on the Watson-Crick face (29).

Finally, our work identifying nuclear and mitochondrial functions for Trm1 in *S. pombe* adds to the growing body of literature-proposing coordination of the nuclear and mitochondrial genomes through tRNA modifications (54). In budding and fission yeast, this may be achieved by altering the balance of transcription of the nuclear- and mitochondrially-targeted Trm1 isoforms, which will in turn influence the degree of G26 dimethylation of nuclear- and mitochondrially-encoded tRNAs. Still, how Trm1-catalyzed tRNA modifications may alter cytoplasmic and mitochondrial translation remains unknown. Although we showed that there were no defects in bulk translation upon deletion of Trm1, we do not exclude the possibility that dimethylation at G26 alters the dynamics of codon recognition, as has been described for methylation of human mitochondrial serine and threonine tRNAs at position 32 by METTL8 (55). Additionally, studying the interplay between Trm1 and other nuclear-encoded enzymes that similarly modify mitochondrial tRNAs will continue to inform our understanding of the control and potential coregulation of the nuclear and mitochondrial genomes.

Our work underscores the complexity of tRNA maturation in a model eukaryote by highlighting the multifunctional nature of a tRNA modification enzyme. The data presented here support the growing idea that tRNA modification enzymes may have roles beyond catalysis and that this holds true in prokaryotes and eukaryotes. The extent to which this applies to other tRNA modification enzymes, and RNA modification enzymes more generally, will be an exciting area of future research that will continue to inform our understanding of the mechanisms underlying RNA fold and function.

## Experimental procedures

### Yeast strains and constructs

Standard laboratory techniques were used to culture *S. pombe* cells at 32 °C. Tag integration and knockouts were generated with a previously described PCR-based strategy and verified by PCR and Western blotting (56) (strains are provided in Table S1). For plasmid-mediated expression of the Trm1 isoforms, the coding sequences of M1 or M24 Trm1 were cloned into the pRep82X yeast expression vector (57). Mutations were introduced by site-directed mutagenesis and verified by sequencing (Table S2). Plasmids were introduced with lithium acetate and selected on minimal media lacking supplements (EMM-ura) (58). tRNA-mediated suppression growth assays were performed as described (34).

### RNA and protein extractions and northern and Western blotting

*S. pombe* cells were grown at 32 °C and harvested at an A_600 nm_ of 0.6. Total RNA was isolated with hot acid phenol, and northern analysis was performed as described using 15% TBE-urea polyacrylamide gels (34). Blots were imaged on a Typhoon FLA 9000 and intensities were quantified with ImageQuant TL software (https://info.cytivalifesciences.com/image-analysis-software.html). For PHA26 northerns (21), relative modification index values represent the intensity of the TUC signal divided by the intensity of the PHA26 signal and normalized to a WT strain or empty vector. For tRNA-mediated suppression northerns, blots were probed with the indicated ^32^P-labeled probes and an equimolar amount of unlabeled competitor probe to prevent hybridization of the labeled probe with the endogenous tRNA Ser^UGA^, as per (16) (probe sequences are provided in Table S3). Total protein was extracted according to (34) and western blots were probed with a custom anti-Trm1 antibody (gift from Dr Richard Maraia, NIH (21)) at 1:2000, β-actin (abcam, ab8226) at 1:1250, and human La/SSB (abcam, ab75927) at 1:1000.

### cDNA synthesis and qRT-PCR

One microgram of Turbo DNase-treated RNA was reverse transcribed with the iScript cDNA synthesis kit (BioRad, 1708890), treated with 0.5 μl RNase cocktail (Invitrogen, AM2286), and diluted 1:10 before quantification using the SensiFAST SYBR No-Rox kit (Bioline, BIO-98005). Quantitative reverse transcriptase polymerase chain reaction (qRT-PCR) was performed with 1 μM of each primer (a common reverse primer for both Trm1 isoforms and isoform-specific forward primers, probes provided in Table S3) with the following qRT-PCR settings: 95 °C for 10 min and 40 cycles consisting of 10 s at 95 °C, 20 s at 60 °C, and 20 s at 72 °C. Trm1 levels were normalized to *act1* mRNA, and normalized M1 Trm1 signal was subtracted from normalized M24 Trm1 signal to calculate relative M24 Trm1 levels.

### Pulse labeling of mitochondrial protein synthesis

Pulse labeling was performed according to (59). Briefly, *S. pombe* cells were initially grown in EMM-ura + 2% glucose, then inoculated into fresh EMM-ura with 0.1% glucose and 2% galactose, and grown at 32 °C for six generations to a final A_600_ less than 1 × 10^7^ cells/ml. Cell pellets corresponding to ∼2.5 × 10^7^ cells were washed with 500 μl reaction buffer (40 mM potassium phosphate pH 6.0, 2% galactose, 0.1% glucose), pelleted, resuspended in 500 μl reaction buffer with 10 mg/ml cycloheximide, and incubated at room temperature for 15 min. Cycloheximide was omitted to evaluate cytoplasmic translation. Five microliters of [^35^S]-methionine was directly added to the cell suspension, mixed thoroughly, and incubated for 30 min at room temperature. Cells were pelleted and the pellet was resuspended in 75 μl solubilization buffer (1.8 M NaOH, 1 M β-mercaptoethanol, 0.01 mM PMSF) and mixed. Five hundred microliters water was added and proteins were TCA-precipitated, followed by separation on a 17.5% SDS gel, transfer to a nitrocellulose membrane, and exposed to a Phosphor screen overnight.

### Recombinant protein purification and EMSA

Recombinant His-tagged protein expression was induced in *Escherichia coli* with 1 mM IPTG at 16 °C for 18 h and purified over a Ni^2+^ column (His-TRAP, GE-Amersham), followed by a second round of purification over a heparin column (Hi-TRAP, GE-Amersham). Proteins were concentrated into 1X EMSA buffer (20 mM Tris–HCl pH 7.6, 100 mM KCl, 0.2 mM EDTA pH 8.0, 1 mM DTT) and quantified by SDS-PAGE (Fig. 5). EMSAs were performed as described (18). Briefly, 3000 cpm PAGE purified, T7-transcribed ^32^P α-ATP-labeled pre-tRNAs (sequences provided in Table S4) were heated to 95 °C and slow-cooled to room temperature before addition to a 20 μL reaction containing 1X EMSA buffer. Increasing amounts of recombinant Trm1 were added to the reaction mix, followed by incubation at 32 °C for 20 min. Reactions were cooled on ice for 2 min and complexes were resolved on 8% nondenaturing polyacrylamide gels run at 4 °C and 100 V. The proportion of bound tRNA (the sum of the initial binding event and any supershifts) was quantified with ImageQuant TL software and binding curves were fit to a nonlinear specific binding curve (specific binding with Hill slope with least squares fit, using the equation Y = B_max_∗X^h^/(K_D_^h^+X^h^, where B_max_ is the maximum specific binding and h is the Hill slope) with GraphPad Prism 10.0.

### *In vitro* methylation and primer extension

Five micromolars of T7-transcribed pre-tRNA Ser^UGA^ was methylated for 3 h at 32 °C in a 25 μl reaction containing 100 mM Tris–HCl pH 7.5, 0.1 mM EDTA pH 8.0, 10 mM MgCl_2_, 40 mM NH_4_Cl_2_, 1 mM DTT, 1.28 mM SAM (NEB, B9003S), and 5 μM or 5 μg recombinant Trm1. For competition between La and Trm1, 5 μM pre-tRNA Ser^UGA^ was pre-incubated alone or with 5 μM recombinant hLa or Sla1 in a 50 μl reaction for 20 min at 32 °C in methylation reaction buffer without MgCl_2_ and SAM. Reactions were snap chilled on ice to preserve complexes, followed by the addition of 5 μM recombinant Trm1, 2 mM MgCl_2_, and 1.28 mM SAM. Reactions were incubated at 32 °C and samples were removed at indicated time points and immediately purified by phenol: chloroform: isoamyl alcohol (25:24:1) extraction. Primer extensions were performed with SuperScript III (Invitrogen, 18080093) following standard methods. For time course reactions, quantifications of G26 modification at each time point were fit to a nonlinear specific binding curve (one-phase association with least squares fit) in GraphPad Prism 10.0 with the equation Y = Y_0_ + (Plateau-Y_0_)∗(1-exp(-K∗X).

### FRET assays

FRET assays were performed as described (17, 41). Briefly, unlabeled and Cy5- and Cy3-labeled RNA substrates were synthesized by IDT (sequences provided in Table S4) and used at a final concentration of 25 nM. Where applicable, recombinant proteins were added to 400 μl reactions at a final concentration of 100 nM immediately prior to taking measurements. Fluorescence emission at 590 and 680 nm was recorded on a Cary Eclipse fluorimeter with readings taken in half-second time-points over a period of 180 s. Strand-dissociation measurements were initiated immediately following strand annealing with the addition of 1 μM unlabeled competitor RNA. Strand annealing and dissociation rate constants were determined by calculating a FRET index (emission at 680 nm divided by emission at 590 nm) over time and normalizing values between 0 and 1. Curves were fitted to an equation for one-phase association (strand annealing) or one-phase decay (strand dissociation) in Graphpad Prism 10.0.

## Data availability

All data pertaining to this manuscript is included in the main manuscript and supporting information.

## Supporting information

This article contains supporting information.

## Conflict of interest

The authors declare that they have no conflicts of interest with the contents of this article.
